# Comparative evaluation of artificial intelligence–assisted literature search tools for identifying clinically meaningful evidence in cardiology

**DOI:** 10.1093/ehjdh/ztag125

**Published:** 2026-07-28

**Authors:** Riccardo Di Febo, Maximiliano Jeanneret Medina, Alexandre Renaud, Maryam Dridi, Valentine Pecriaux, Benoit Lequeux, Stephane Lafitte, Baptiste Maille, Aymeric Menet

**Affiliations:** Department of Cardiology, Hôpital Saint Philibert, Groupement des Hôpitaux de L'Institut Catholique de Lille, Université Catholique de Lille, 115 Rue du Grand But, 59160 Lille, France; Laboratoire ETHICS, DataCœur Research Team, Groupement des Hôpitaux de L’Institut Catholique de Lille, 115 Rue du Grand But, 59160 Lille, France; Division of Cardiology, Department of Health Sciences, San Paolo Hospital, University of Milan, Via Antonio di Rudinì, 8, 20142 Milan, Italy; HEG Arc, HES-SO, University of Applied Sciences Western Switzerland, 2000 Neuchâtel, Switzerland; EM Normandie Business School, Métis Lab, 30-32, Rue Henri-Barbusse, Clichy 92110, France; Department of Cardiology, Hôpital Saint Philibert, Groupement des Hôpitaux de L'Institut Catholique de Lille, Université Catholique de Lille, 115 Rue du Grand But, 59160 Lille, France; Laboratoire ETHICS, DataCœur Research Team, Groupement des Hôpitaux de L’Institut Catholique de Lille, 115 Rue du Grand But, 59160 Lille, France; Department of Cardiology, Hôpital Saint Philibert, Groupement des Hôpitaux de L'Institut Catholique de Lille, Université Catholique de Lille, 115 Rue du Grand But, 59160 Lille, France; Laboratoire ETHICS, DataCœur Research Team, Groupement des Hôpitaux de L’Institut Catholique de Lille, 115 Rue du Grand But, 59160 Lille, France; Cardiology Department, University Hospital of Poitiers, 2 Rue de la Milétrie, Poitiers F-86021, France; Bordeaux University, Department of Cardiology, Bordeaux University Hospital, Bordeaux F-33000, France; Department of Cardiology, Marseille University Hospital Timone, 13005 Marseille, France; Department of Cardiology, Aix-Marseille University, 13005 Marseille, France; Center for CardioVascular and Nutrition Research (C2VN), INSERM, INRA, 13005 Marseille, France; Department of Cardiology, Hôpital Saint Philibert, Groupement des Hôpitaux de L'Institut Catholique de Lille, Université Catholique de Lille, 115 Rue du Grand But, 59160 Lille, France; Laboratoire ETHICS, DataCœur Research Team, Groupement des Hôpitaux de L’Institut Catholique de Lille, 115 Rue du Grand But, 59160 Lille, France

**Keywords:** Artificial intelligence, Digital health, Cardiac resynchronization therapy, Literature search

## Abstract

**Aims:**

The rapid expansion of biomedical literature challenges clinicians’ and researchers’ ability to identify clinically meaningful evidence. We systematically compared five literature search tools, four artificial intelligence (AI)-assisted and one conventional, across clinically relevant cardiology research scenarios, using a blinded expert-validated gold standard to assess their ability to retrieve relevant and key references.

**Methods and results:**

We evaluated ChatGPT-5, Elicit, Consensus, Scite, and PubMed across four cardiology topics defined by maturity and specificity, with multiple standardized prompts. Three electrophysiology experts independently and blindly rated all retrieved references, defining two gold standards: expert-rated relevance and expert-selected key references. ChatGPT-5 achieved the highest proportion of relevant articles (90% [88–100], *P* < 0.001) and the highest key-reference overlap (60% [43–68], *P* < 0.001), whereas Scite performed lowest (20% and 10%, respectively). The tool was the primary determinant of performance (partial *R*^2^ = 0.50), whereas prompt formulation had no significant effect. In a pre-specified subanalysis restricted to clinical studies, ChatGPT-5 and human-conducted systematic reviews overlapped by 42% (96% of shared articles highly relevant), with 58% distinct references, indicating complementary AI and human retrieval; ChatGPT-5 produced hallucinated citations when long reference lists were requested for emerging topics, underscoring the need for human verification.

**Conclusion:**

AI-assisted tools showed heterogeneous performance, ChatGPT-5 performing best in this cardiology setting. These preliminary, context-specific findings support hybrid human–AI strategies in which AI complements rather than replaces transparent database searches such as PubMed; larger-scale, multi-domain studies are needed to confirm and generalize them.

## Graphical Abstract

**Graphical Abstract:**
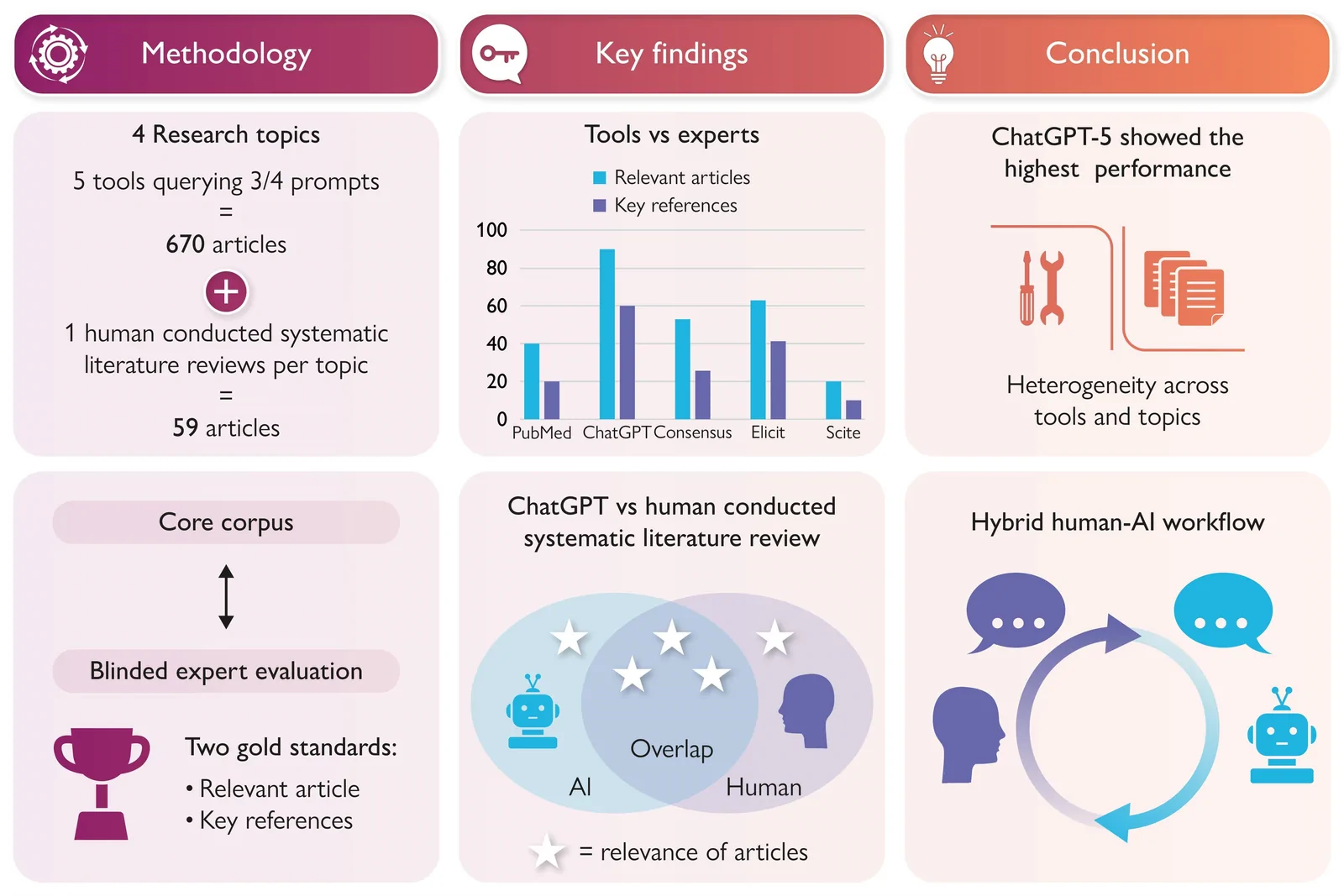

## Introduction

The rapid expansion of biomedical publications has made it increasingly difficult to retrieve reliable evidence. This information overload widens gaps in search proficiency and threatens the reproducibility of literature-based research. Although systematic reviews remain the most rigorous method for evidence synthesis, they require advanced search skills and considerable time, resources that are often limited in routine clinical and translational research settings. As a result, evidence retrieval has become a critical bottleneck in evidence-based medicine, with important implications for reproducibility, guideline development, and clinical decision-making.

Recent advances in artificial intelligence (AI), particularly large language models (LLMs) and AI-assisted literature search platforms, have emerged as potential solutions to streamline evidence retrieval.^1,2^ These tools promise to lower technical barriers by enabling natural-language querying, semantic expansion, automated ranking, and rapid summarization of the literature,^3,4^ with growing interest in their application across cardiovascular medicine.^5,6^ However, concerns persist regarding hallucinations, incomplete source coverage, temporal inconsistencies, and substantial variability in distinguishing between high- and low-quality studies.^4,7^ Importantly, few evaluations have benchmarked AI-driven retrieval against expert-validated systematic reviews within real-world clinical domains. Furthermore, empirical evaluations have been mostly focused on assessing one tool applied to a single topic.^1^

Cardiac resynchronization therapy (CRT) provides an ideal evaluation setting for several reasons. First, it encompasses both established and emerging research questions, spanning landmark randomized controlled trials (RCTs) from the early 2000s to contemporary investigations of conduction system pacing and AI-guided patient selection. Second, the CRT evidence base includes multiple high-quality systematic literature reviews (SLRs) that can serve as domain-specific gold standards. Third, CRT exemplifies a field where incomplete evidence retrieval has direct clinical consequences, as eligibility criteria and device selection depend critically on up-to-date knowledge of trial data and guideline recommendations. In this study, we systematically compare five literature search tools (ChatGPT-5, Elicit, Consensus, Scite, and PubMed) across clinically realistic research scenarios, using a core corpus derived from blinded expert evaluation. This study aimed (i) to evaluate the ability of each tool to retrieve relevant and key articles across research topics, and (ii) to assess the added value of AI-assisted retrieval compared with human-driven searches.

## Methods

### Study design overview

**Figure 1:**
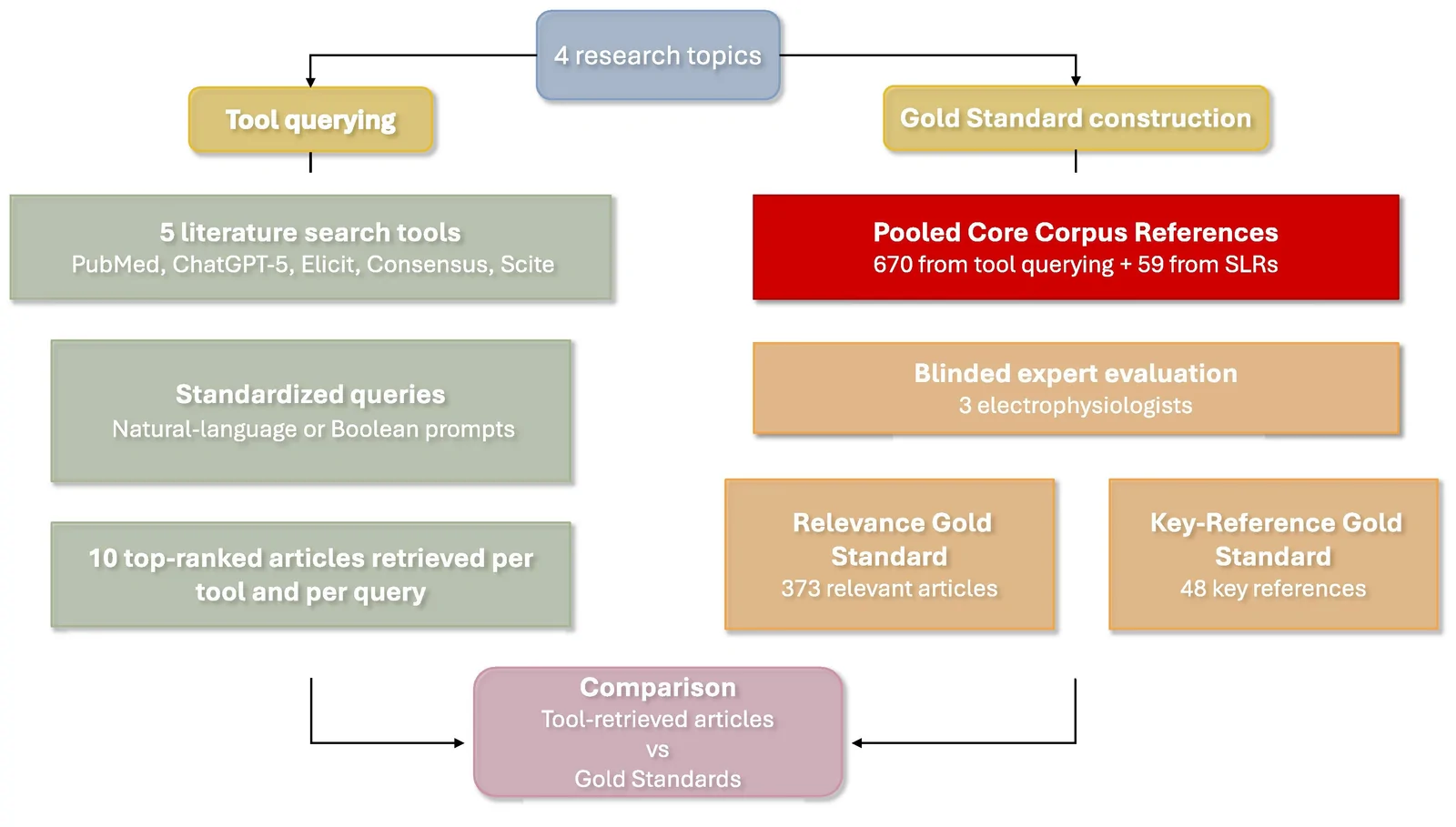
Methodological workflow of the study. Five literature search tools, four AI-based (Elicit, Consensus, Scite, ChatGPT-5) and the conventional Boolean database PubMed, were evaluated across four cardiology research topics defined by maturity and specificity. Each tool was queried with standardized queries (four natural-language prompts per AI-based tool; three Boolean queries of increasing complexity for PubMed), retrieving up to 10 top-ranked articles per tool and per query. The tool-retrieved references were pooled with the 59 primary references of the highest-quality systematic literature reviews (SLRs), one SLR per topic, to form the core corpus, which three electrophysiology experts assessed independently and in a blinded fashion. Two complementary gold standards were derived: a *relevance gold standard* (articles with a mean expert relevance score >2.5 on a 0–3 scale) and a *key-reference gold standard* (for each topic, the 10 most relevant original studies plus the most relevant systematic review and meta-analysis). Each tool’s retrieved articles were then compared against both gold standards. Counts shown are tool-retrieval records (an article retrieved by several tools for the same topic is counted once per tool); after within-topic deduplication the core corpus comprises 342 unique article-topic combinations and the relevance gold standard 118 unique article-topic combinations, as reported in the Results. A fully detailed version of this workflow, including the source-to-standard breakdown, is provided in the Supplementary material (Supplementary material online, *Figure S1*).

The methodological workflow is summarized in *Figure 1*. We systematically evaluated five literature search tools (ChatGPT-5, Elicit, Consensus, Scite, and PubMed) across four clinically relevant cardiology research topics defined by their maturity and specificity. Multiple standardized prompts were tested. For each tool, topic, and prompt, we selected the top ten ranked articles whenever possible. Our inter-tool and inter-condition comparison relies on offline evaluation, a widely used method in scientific document retrieval which aims to find papers that meet a user's needs as expressed in a query.^8,9^

To do so, and following recent studies with a comparable objective to ours,^1,10^ we established two complementary gold standards: Expert-selected key references and expert-rated relevance. These reference sets were constructed through blinded, independent expert assessment of all retrieved articles, including references originating from high-quality SLRs and those identified by the evaluated search tools.

### Tools

We evaluated five tools: ChatGPT-5, a general-purpose LLM; Elicit, Consensus, and Scite, three of the most widely used AI-based literature review tools; and PubMed, the conventional Boolean-based biomedical database.^11–14^ All AI-assisted tools were accessed through their publicly available web interfaces (free tier) between August 2025 and January 2026; no API access was used, and no advanced, paid, or custom configuration (e.g. temperature, custom instructions) was applied. Live online retrieval was enabled for every tool: for ChatGPT-5, the web-search function was available, so that responses could be grounded in real-time web retrieval rather than parametric memory alone; Elicit and Consensus retrieve by construction from their indexed scholarly corpora (Semantic Scholar, OpenAlex, and PubMed coverage), using semantic and hybrid semantic/keyword (BM25) search respectively; and for Scite Assistant the source setting was configured to supplement its citation database with the open web. ChatGPT-5 was queried using the GPT-5 model; for all tools, each prompt was submitted in a separate conversation with history cleared between queries, and ChatGPT-5’s cross-session memory was inactive, so that no information from previous queries could influence subsequent responses. The technical characteristics of all five tools are summarized in Supplementary material online, *Table S1* in the Supplementary material.

### Operationalization of retrieval tools

All references retrieved by the five tools were manually verified by the investigators to confirm bibliographic accuracy (publication title, authors, journal, year of publication, digital object identifier (DOI), study design, impact factor of journal, citation count). From a clinical-utility perspective, we distinguished two categories of citation inaccuracy. Citations containing minor bibliographic discrepancies (errors in title, authors, journal, or DOI) for which the intended source article could nonetheless be reliably identified and retrieved were considered locatable, corrected, and not classified as hallucinations, since the underlying evidence remains accessible to the clinician. Conversely, citations that could not be traced to any identifiable source after DOI and title searches were classified as hallucinated (fully fabricated), as the cited evidence does not exist and can neither be retrieved nor verified.

### Research queries and search quadrants

**Figure 2:**
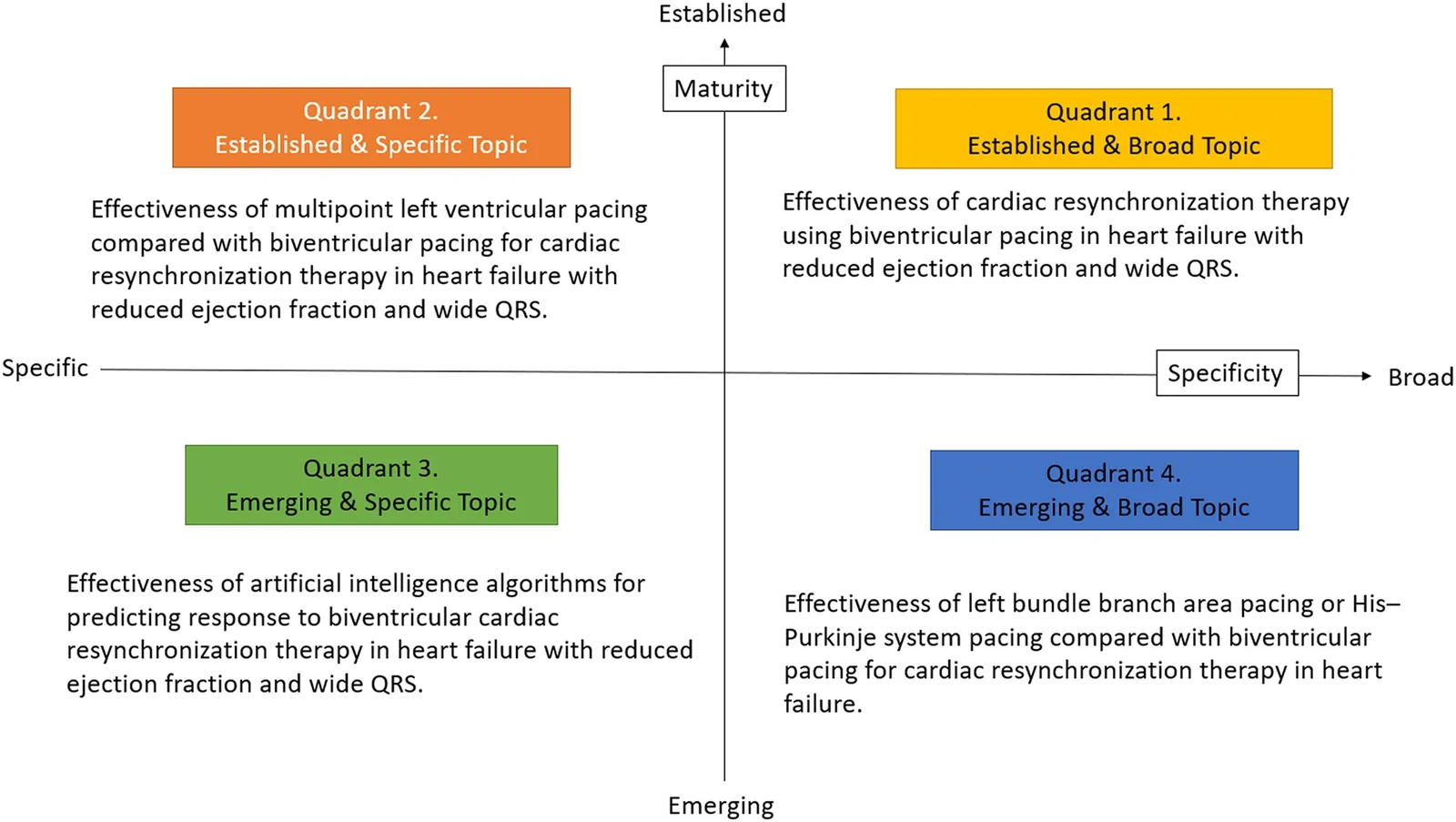
Illustration of the four research quadrants used in this study, defined by the maturity of the topic (established vs. emerging) and the specificity of the research question (broad vs. specific).

Four research questions were defined along two dimensions: query specificity (broad vs. specific) and topic maturity (established vs. emerging), thus forming the four search quadrants described in the scientometric literature^15,16^ (*Figure 2*).

For each AI-based tool and each question, four natural-language prompts were designed to reflect typical clinical use cases: research oriented, practice oriented, general questioning and active form (focus on the literature review task). For ChatGPT-5, a specific prompt section was added to provide ten scientific references in APA format with DOI. Each prompt was submitted once per tool and topic; repeated runs of the same prompt were not performed.

For PubMed, three Boolean queries of increasing complexity were developed, ranging from simple keyword combinations to expert-level structured search strategies (*Table 1*, Supplementary material online, *Table S2* in the Supplementary material).

**Table 1:** Example of queries for topic a (developed and broad topic)

***Natural language tools***	
**Research oriented**	How effective is cardiac resynchronization therapy by biventricular pacing in low ejection fraction heart failure with wide QRS?
**Practice oriented**	What is the clinical benefit of cardiac resynchronization therapy by biventricular pacing in low ejection fraction heart failure with wide QRS?
**General questioning**	What are the impacts of cardiac resynchronization therapy in heart failure?
**Active form (task)**	Find me accurate references about effectiveness of cardiac resynchronization therapy by biventricular pacing in low ejection fraction heart failure with wide QRS
***PubMed***	
**Boolean**	(‘cardiac resynchronization therapy’) AND ((‘heart failure’) OR (‘cardiac failure’))
**Expert**	(‘cardiac resynchronization therapy’ OR ‘biventricular pacing’ OR ‘CRT’) AND (‘heart failure’ OR ‘congestive heart failure’) AND (‘effectiveness’ OR ‘efficacy’ OR ‘outcome’ OR ‘mortality’) AND ((‘N Engl J Med’ [Journal]) OR (‘Lancet’[Journal]) OR (‘JAMA’ [Journal]) OR (‘Circulation’[Journal]) OR (‘Eur Heart J’ [Journal]) OR (‘J Am Coll Cardiol’ [Journal])) AND (‘1900/01/01’ [Date - Publication] : ‘2006/12/31’ [Date - Publication]) AND (randomised controlled trial[pt])
**Simple**	Cardiac resynchronization therapy heart failure effectiveness

Categories of natural-language prompts used to query each tool, reflecting four typical clinician use cases: research-oriented, practice-oriented, general inquiry, and active prompts focused on the literature-review task. PubMed queries were reformulated using keywords and Boolean operators to match its search syntax. The phrase: ‘Provide ten scientific references in APA format with DOI for each document.’ was added to ChatGPT-5 prompts in order to be comparable with dedicated AI-based literature-review tools. The prompts used for the three remaining topics are detailed in Supplementary material online, *Table S2* in the Supplementary material.

### Gold-standard development and expert validation

Two complementary sources were used to establish the gold standard and to assess the quality of the corpus of articles proposed by the five tools.

First, we identified the highest-quality SLR for each topic and isolated their primary references that we consider as the human reference standard. They were selected based on methodological rigour, including systematic review or meta-analysis design, PRISMA-compliant reporting, publication in high-impact cardiology or internal medicine journals, and alignment with predefined research themes. Second, we compiled all the references retrieved by the five evaluated tools using the predefined prompts and queries.

Then, three electrophysiology experts independently reviewed all retrieved references, establishing two gold standards:

- *Relevant articles gold standard.* Each article was rated using a relevance scale (0 = not relevant, 1 = marginally relevant, 2 = relevant, 3 = highly relevant). Articles with a mean relevance score greater than 2.5 were considered clinically relevant and included in the relevance gold standard.
- *Key references gold standard.* For each topic, experts also identified a set of key references representing the highest-quality evidence, consisting of the 10 most relevant primary studies (top 10), the most relevant systematic review, and the most relevant meta-analysis.

Both primary metrics were precision-type measures, computed as the proportion of each tool’s retrieved articles that were, respectively, rated as clinically relevant or identified as expert-defined key references.

For each topic, all references retrieved by the five tools, together with the primary references of the selected SLRs, were aggregated into a single pooled core corpus. Because relevance was assessed relative to each research question, deduplication was performed within topics: an article retrieved by several tools for the same topic was reconciled on an article-by-article basis and rated only once, whereas the same article appearing under two topics was treated as two distinct units and could be rated relevant for one topic but not the other. Retrieval credit was nonetheless attributed independently to every tool that had returned it. The three experts assessed this corpus independently and asynchronously, blinded to the originating tool or source, thereby preventing both mutual influence and tool-related bias. They rated only the articles contained in the corpus and could not add references of their own, so that both gold standards were drawn exclusively from this predefined corpus.

### Other measurements

For each retrieved article, the following characteristics were compiled and analysed: clinical condition, journal of publication, full bibliographic reference, DOI, study design (RCT, observational study, meta-analysis, guideline, or other), journal impact factor, year of publication, number of patients, and citation count. These variables were used to characterize the type, visibility, and clinical relevance of the evidence identified by each tool.

### Comparison with human-conducted SLRs

AI-assisted tools retrieved a broader range of article types, whereas SLRs, by definition, include only predefined clinical studies. As a result, this methodological difference may underestimate the apparent performance of AI-assisted tools when compared with SLR-derived reference standards.

To directly compare the recall performance of AI-assisted retrieval with that of human-conducted SLRs, we performed a predefined subanalysis restricted to original clinical studies (RCTs and observational cohort studies). ChatGPT-5 was asked to retrieve the same number and type of articles as those included in the reference SLR for each topic (topic A: 14 articles; topic B: 11 articles; topic C: 22 articles; topic D: 12 articles) (see Supplementary material online, *Table S3*).

The prompt used was identical to that of the main analysis, with the addition of the following instruction: ‘Provide a list of [number] references in APA format with DOI, including only clinical trials and cohort studies (excluding meta-analyses, systematic reviews, and narrative reviews), published up to [SLR reference date].’

This approach enabled a controlled comparison between AI-assisted retrieval and human systematic review processes, ensuring equivalent scope in terms of study design, number of articles, and temporal coverage. Only one AI tool was used in this subanalysis to ensure methodological consistency and because it had demonstrated the highest performance in the primary evaluation.

### Statistical analysis

Quantitative data are presented as mean ± standard deviation or median [25th–75th percentile] in case of skewness. Qualitative data are presented as absolute numbers and percentages.

Differences between groups were assessed using the Kruskal–Wallis test for continuous variables and the χ^2^ test for categorical variables.

Inter-rater agreement among the three independent experts was assessed using Fleiss’ kappa coefficient for categorical ratings. Agreement was evaluated separately for relevance classification and identification of key references.

To evaluate differences in the percentage of relevant articles and key references across topics and tools, two multivariable linear regression models were constructed with the percentage of relevant results and key reference as the dependent variables and tool and topic as independent variables.

The assumptions of the linear regression models (residual normality and homoscedasticity) were assessed using the Shapiro-Wilk and Breusch-Pagan tests. To account for repeated measurements across prompts within each tool-topic combination, both models were additionally refitted as linear mixed-effects models with a random intercept for prompt, as a sensitivity analysis. For all tests, a two-tailed *P* value of 0.05 or less was considered statistically significant. Statistical analysis was performed with R 4.3.0 (Youngstown, Ohio, USA).

## Results

### Corpus of articles

Across the four topics, the five tools returned 670 eligible references after applying the temporal inclusion criteria (PubMed 105; ChatGPT-5 154; Consensus 158; Elicit 140; Scite 113; *Table 2*). These numbers were not identical across tools because the tools did not always return ten articles per query. After deduplication across tools, these corresponded to 316 unique article-topic combinations, indicating substantial overlap among the tools’ retrievals. The four human-conducted SLRs contributed 59 primary references, of which 33 had also been retrieved by at least one tool and 26 by none. Pooling the tool-retrieved and SLR references therefore yielded a corpus of 342 unique article-topic combinations, each independently reviewed and rated once, in a blinded fashion, by three experts.

**Table 2:** Comparative descriptive analysis of the sample

	All tools	PubMed	ChatGPT-5	Consensus	Elicit	Scite	*P*-value
**Number of articles**	670	105	154	158	140	113	
**Citations**	93 [24;280]	31 [9 ;169]	130 [49;513]	102 [31;306]	97 [17;280]	45 [21;246]	<0.001
**Impact factor**	7.4 [2.9;21.7]	5.3 [2.3;10.8]	7.7 [5.7;21.7]	7.4 [5.7;22.3]	7.4 [3.1;22.3]	5.9 [2.3;10.8]	<0.001
**Years difference from reference**	3.4 [1.9;4.9]	3.4 [1.9;4.9]	3.2 [2.4;4.4]	2.9 [1.4;5.4]	2.92 [1.42;5.42]	4.4 [2.9;7.4]	0.002
**Number of patients**	180 [44;595]	110 [44;804]	381 [50;752]	176 [44;406]	306 [44;668]	161[50;758]	0.097

### Descriptive characteristics of retrieved articles

**Figure 3:**
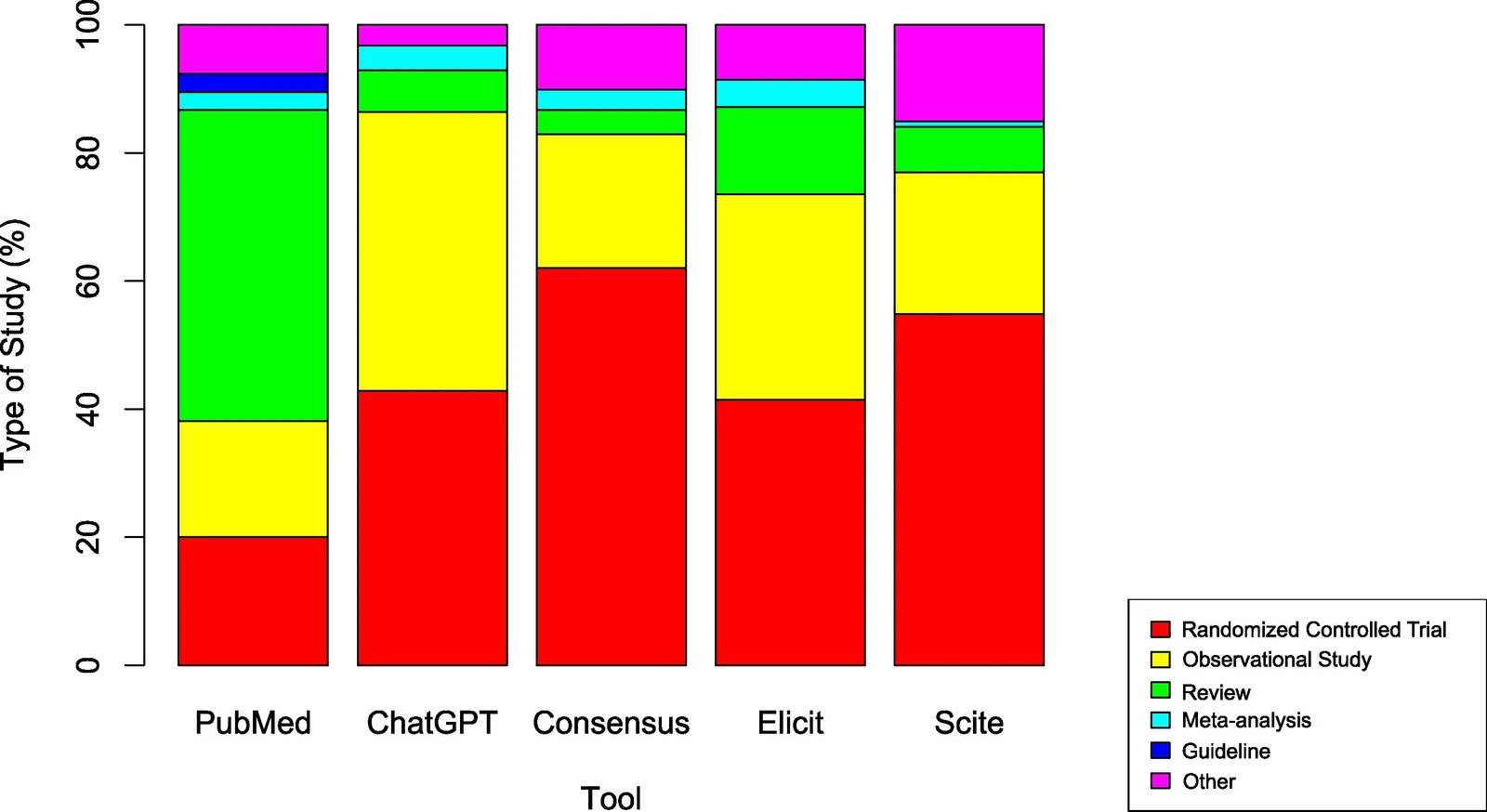
Types of studies among articles retrieved by tools.

Descriptive analyses are summarized in *Table 2* and *Figure 3*.

ChatGPT-5 retrieved articles with the highest median citation count (130 [49–513]). ChatGPT-5, Consensus, and Elicit identified articles published in journals with higher median impact factors. Scite retrieved older articles than other tools (4.4 [2.9–7.4] years) and consistently underperformed across descriptive metrics. PubMed retrieved the highest proportion of review articles (38%), whereas AI-assisted tools retrieved nearly twice as many original articles as PubMed.

### Relevance of retrieved articles

Following expert evaluation, ChatGPT-5 demonstrated the highest proportion of expert-rated relevant articles (90% [88–100], *P* < 0.001. The highest relevance proportions were observed for topic A (100% [95–100]) and topic C (95% [90–100]), where ChatGPT-5 retrieved a higher proportion of relevant articles than the other evaluated tools.

**Figure 4:**
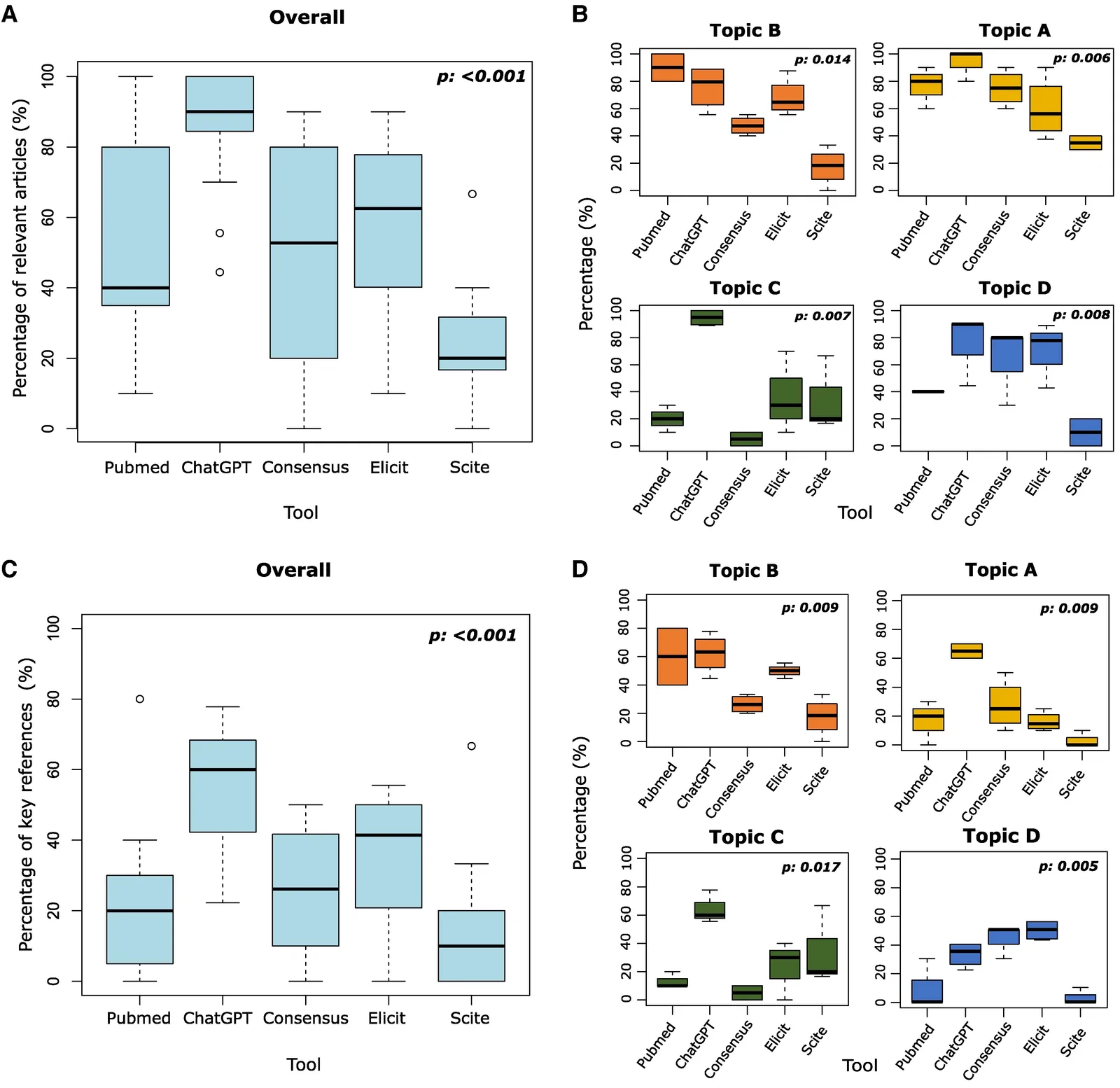
(*A*) Overall performance and (*B*) performance by research topic of proportion of relevant articles retrieved by tools. (*C*) Overall performance and (*D*) performance by research topic of proportion of expert-defined key reference articles retrieved by tools.

Conversely, Scite showed the lowest relevance, with only 20% [17–31] of articles deemed pertinent. Other tools showed intermediate performance. Notably, PubMed achieved the highest relevance for topic B (90% [85–95]) (*Figure 4, A-B*).

The agreement between the three experts was substantial (κ = 0.72, 95% confidence interval [CI] 0.67–0.78).

In the multivariable model, the retrieval tool was the main determinant of relevance rates, accounting for 50% of the explained variance (partial *R*^2^ = 0.50). Compared with PubMed, ChatGPT-5 retrieved a significantly higher proportion of relevant articles (β = + 32 [15;49] %, *P* < 0.001), whereas Scite showed significantly lower performance (β = −31 [−48; −13] %, *P* < 0.001). Topic C was associated with a significantly lower relevance rate compared with topic A, B, and D (see Supplementary material online, *Table S4*).

### Key references retrieval

Regarding key references, ChatGPT-5 showed the highest overall performance, retrieving 60% [43–68] of expert-defined key reference articles. Topic-specific performance was highest for topic A, topic B, and topic C. In contrast, Scite demonstrated the lowest performance, retrieving only 10% [0–20] of key references (*Figure 4, C-D*).

Inter-rater agreement was substantial (κ = 0.7, 95% CI 0.61–0.77).

In the multivariable linear regression model, the retrieval tool was the main determinant of the rate of key reference retrieval, explaining 44% of the model variance (partial *R*^2^ = 0.44). Compared with PubMed, ChatGPT-5 retrieved a significantly higher proportion of key references (β = + 33%, 95% CI 19–47; *P* < 0.001). Topic B was associated with a higher key reference rate retrieval compared with topic A, C, and D (see Supplementary material online, *Table S5*). Residual diagnostics supported the two models, and a mixed-effects model with a random intercept for prompt yielded essentially unchanged estimates, confirming robustness to the repeated structure of the data.

### Prompt analysis

No statistically significant differences in retrieval performance were observed according to prompt formulation across tools (see Supplementary material online, *Figure S2* and *Figure S3*). Among AI-assisted tools, variations in natural language prompts did not significantly affect relevance or key reference retrieval. Similarly, increasing Boolean query complexity in PubMed did not significantly improve performance.

### Comparison between ChatGPT-5 and human-conducted SLRs

**Figure 5:**
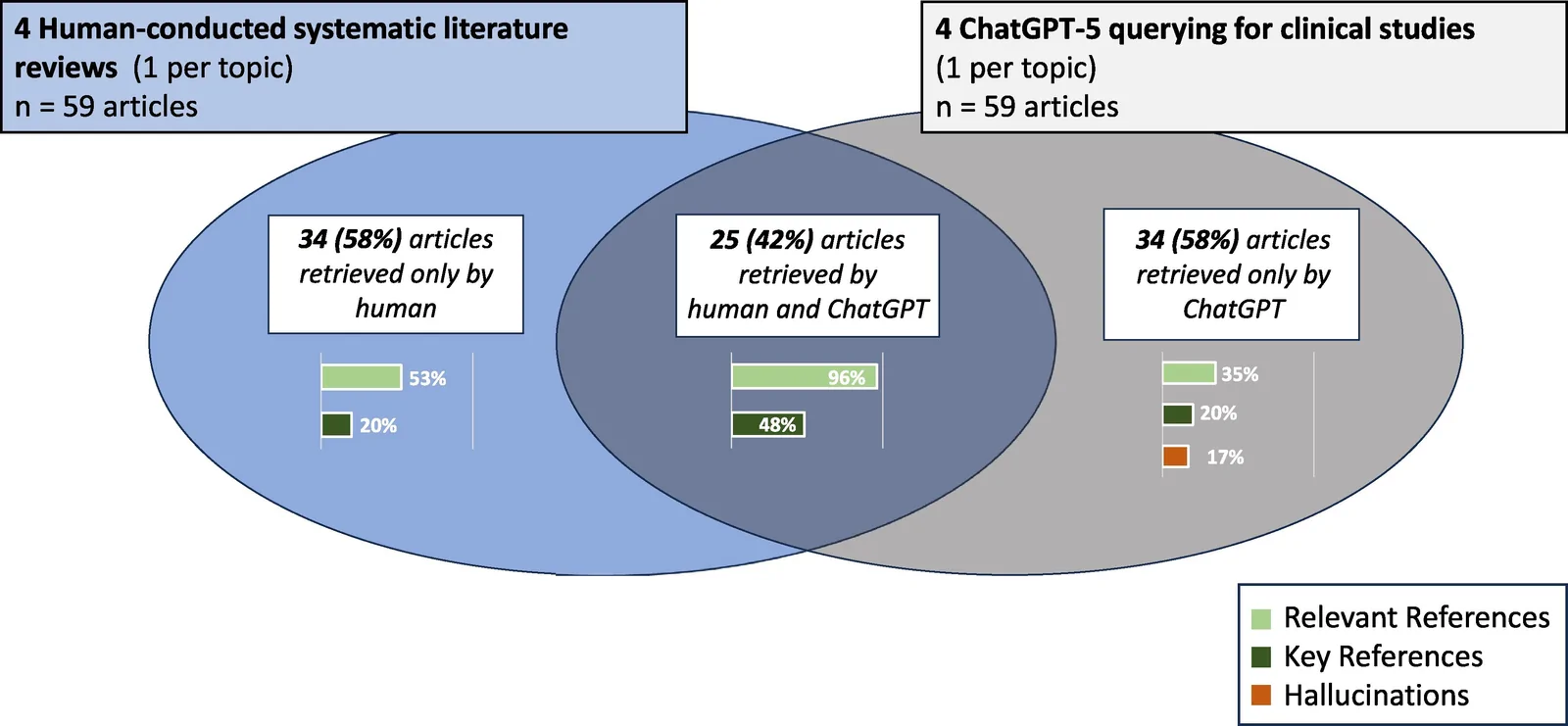
Comparison between ChatGPT-5 vs. human-conducted systematic literature reviews.

In this predefined subanalysis restricted to clinical studies, 25 articles (42%) were retrieved by both ChatGPT-5 and the human-conducted SLRs. Among these overlapping articles, 24 (96%) were rated as highly relevant and 12 (48%) were identified as expert-defined key references (*Figure 5*).

Conversely, 34 articles (58%) included in the SLRs were not retrieved by ChatGPT-5. Of these, 18 (53%) were rated as relevant and 7 (20%) were classified as key references. ChatGPT-5 also identified 34 additional articles that were not included in the SLRs, of which 12 (35%) were rated as relevant and 7 (20%) were considered key references.

### Citation accuracy and hallucinated references

In the main analysis (up to ten references per condition), no hallucinated citations occurred across any tool, topic, or prompt; only minor, correctable discrepancies in titles, DOIs, or author names arose, and their source articles were reliably located. Fabricated, untraceable citations were confined to the clinical-studies subanalysis, which requested larger lists: ChatGPT-5 produced ten (17%), nine of them in topic C, an emerging, broad topic requiring the longest list (22 articles), one in topic D, and none in the established topics, all beyond the 11th reference. Hallucinations thus rose in emerging topics and with the number of references requested, but were absent within the realistic range of about 10 references per query.

## Discussion

In this systematic, expert-validated evaluation of AI-assisted literature search tools in clinically relevant cardiology scenarios, performance varied substantially across tools. Within the four cardiology topics evaluated, ChatGPT-5 showed the highest performance for both overlap with expert-defined key references and proportion of relevant articles. These differences likely reflect variations in underlying retrieval architectures, database coverage, and ranking algorithms, and align with recent work showing that retrieval-augmented language models improve citation accuracy compared with standalone models.^17^ These results should be interpreted as preliminary, context-specific observations rather than as evidence of general superiority. A central methodological contribution of this study is the use of a dual expert-validated gold standard combining relevance ratings and key reference identification, constructed through blinded independent assessment. The substantial inter-rater agreement observed (κ=0.70–0.72) supports the robustness of our benchmarks and strengthens the validity of inter-tool comparisons. Multivariable analyses confirmed that the retrieval tool was the primary determinant of performance, explaining 44–50% of variance, whereas the influence of the research topic was more limited. This finding has practical implications: tool selection should be an explicit methodological decision rather than a matter of convenience. Notably, PubMed, despite its conventional Boolean architecture, achieved competitive performance for specific topics, underscoring the continued relevance of structured database search in well-indexed research domains.

Variations in query formulation did not significantly affect retrieval performance across tools, suggesting that modern AI retrieval systems are sufficiently robust to interpret varied natural-language inputs consistently. This observation is reassuring for clinicians without expertise in Boolean search strategies and challenges the common assumption that prompt engineering is a critical determinant of AI retrieval quality.

A key observation of this study is the complementarity between AI-assisted and human systematic searches. In the clinical studies subanalysis, ChatGPT-5 and human-conducted SLRs shared only 42% of articles, while each identified a substantial proportion of relevant and key references not captured by the other. Notably, among the 25 articles retrieved by both approaches, 96% were rated as highly relevant by independent experts, suggesting that convergence between AI-assisted and human searches may serve as a strong signal of evidence quality. The 34 articles identified exclusively by ChatGPT-5, of which 35% were rated as relevant and 20% as key references, further suggest that AI-assisted tools may broaden retrieval beyond the scope of predefined keyword strategies, offering complementary value even when compared with rigorously conducted systematic reviews.

However, the reliability of AI-generated outputs remains a concern. In the subanalysis, 17% of citations were fabricated, concentrated in the emerging, broad topic and only beyond the eleventh reference, suggesting that fabrication arises when the number of requested references exceeds the genuinely available evidence. Notably, this fabrication rate is lower than the citation-fabrication rates reported for earlier, ungrounded language models.^18^ This most likely reflects the use of web-search grounding, which anchors generated citations in retrievable sources rather than in parametric memory, consistent with evidence that retrieval-augmented models improve citation accuracy.^17^ AI-generated reference lists should therefore be restricted to a small number of top-ranked citations and subjected to systematic expert verification, particularly for emerging or rapidly evolving research topics.^19^ Moreover, the structured formatting of AI-generated citations, complete with journal names, volumes, and DOIs, may increase their perceived credibility and discourage verification, amplifying the risk of inadvertent propagation of erroneous references in downstream literature. Consistently, recent experimental data show that citation fabrication by LLMs varies systematically with topic specificity and scientific maturity, underscoring the importance of verification workflows tailored to the research domain.^19^

Taken together, these results support a hybrid model in which AI-assisted tools augment rather than replace transparent database searches and expert-driven methods. AI-assisted retrieval is particularly useful for exploratory searches and hypothesis generation, but ChatGPT-5’s higher performance does not justify standalone use, given risks of opaque source coverage, retrieval bias, and unverifiable citations. Conversely, PubMed and citation-context platforms such as Scite, though narrower, offer grounded, traceable, and reproducible sources. ChatGPT-5 is therefore best used as a complementary tool requiring expert validation.

This study has several limitations. First, the evaluation was restricted to four research questions within a single, relatively narrow subdomain of cardiology (CRT) and relied on a predefined set of standardized prompts; while these choices ensured methodological control, they limit the generalizability of our findings to other clinical fields, broader research questions, and unstructured real-world queries. Our results should therefore be regarded as preliminary, hypothesis-generating evidence specific to this context rather than as a general demonstration of tool superiority. In addition, because large language models are non-deterministic, identical prompts may yield different outputs across runs; as each prompt was submitted only once, we did not quantify this run-to-run variability or the test–retest reliability of retrieval. Tool performance was assessed at a single time point, and the rapidly evolving nature of AI models means findings may not remain stable across software updates or model versions. All tools were evaluated in freely available versions, and performance may differ in premium tiers. We also acknowledge a potential incorporation bias, since the evaluated tools contributed to the gold-standard corpus. This was inherent to our aim, as assessing the added value of AI-assisted retrieval requires rating tool-retrieved articles beyond the SLR set. We mitigated it through independently selected SLR references, blinded assessment, and precision-type metrics. The pre-specified ChatGPT-5-vs.-SLR subanalysis (*Figure 5*) already provides a sensitivity analysis against a predefined benchmark independent of the tools; extending this to multiple clinical domains would further strengthen the conclusions. A further limitation concerns the heterogeneity in database coverage across platforms. Part of the observed performance differences may therefore reflect disparities in indexed literature rather than retrieval or ranking capability, which our design cannot disentangle. Moreover, the evaluated tools differ in their intended purpose, such that the observed differences reflect a comparison between functionally distinct categories of tools rather than a uniform performance ranking; each tool’s performance should therefore be construed as that of an integrated system encompassing its corpus, ranking strategy, and intended use. Finally, although expert evaluation strengthened the analysis, expert judgement remains inherently subjective despite blinded assessment.

In conclusion, AI-assisted literature retrieval tools demonstrated heterogeneous performance, with ChatGPT-5 achieving the highest retrieval accuracy among the evaluated tools across most metrics in this specific cardiology context. The complementarity between AI-assisted and human systematic searches supports a hybrid retrieval model, with human oversight remaining essential to ensure citation accuracy and methodological rigour. Larger-scale studies spanning multiple clinical domains and more diverse, real-world query types are needed to confirm and generalize these findings and to inform best-practice guidelines for integrating AI tools into evidence-based clinical research workflows.

## Supplementary Material

ztag125_Supplementary_Data

## Data Availability

Complete prompts list and SLRs references are available in the Supplementary material, while access to complete core corpus will be granted on reasonable request to the corresponding author (menet.aymeric@ghicl.net), following a formal data-use request process.

## Lead author biography

**Riccardo Di Febo, MD**, is a cardiology resident at the San Paolo Hospital, University of Milan, Italy, and a cardiac electrophysiology fellow at the Saint Philibert Hospital, Groupement des Hôpitaux de l'Institut Catholique de Lille (GHICL), Université Catholique de Lille, France, where he is a member of the DataCœur Research Team (Laboratoire ETHICS). His research focuses on the intersection of artificial intelligence and cardiovascular medicine, atrial fibrillation catheter ablation, cardiac resynchronization therapy and conduction system pacing.
