# Supplementary material for: Comparative evaluation of artificial intelligence–assisted literature search tools for identifying clinically meaningful evidence in cardiology
Source: Eur Heart J Digit Health. 2026 Jul 28;7(7):ztag125. doi: 10.1093/ehjdh/ztag125 (PMC13520940; doi:10.1093/ehjdh/ztag125)
Supplement: Supplementary file 1 [file ztag125_supplementary_data.docx]

**Supplementary Appendix**

**Supplementary Materials**

Table of Contents

Supplementary Tables ……………………………………..... 3

Supplementary Figures ……………………………………. 11

**Supplementary Tables**

| **Tool** | **Type** | **Version** | **Access** | **Corpus** | **Retrieval** | **Web retrieval** | **Configuration** | **Query type** |
| --- | --- | --- | --- | --- | --- | --- | --- | --- |
| **PubMed** | Conventional Boolean biomedical database | Continuously updated (NLM/NCBI) | Public web interface | MEDLINE/PubMed (~40M biomedical citations) | Boolean / MeSH exact-match keyword search | Source database | 3 Boolean queries (simple, Boolean, expert) | Boolean queries |
| **ChatGPT-5** | General-purpose large language model | GPT-5 model (released Aug 7, 2025) | Web interface, free tier | Parametric knowledge + live web search | LLM generation grounded by web search; unified “router” architecture (fast vs reasoning) | Web search available | Default; no parameter changes; cross-session memory inactive; new conversation per prompt | 4 natural-language prompts + specific instruction |
| **Elicit** | AI literature-review assistant | Freely available version at the time | Web interface, free tier | Semantic Scholar, OpenAlex, PubMed, ClinicalTrials.gov (over 125M papers) | Retrieval-augmented semantic search | Native retrieval over indexed scholarly corpus | Default; new conversation per prompt | 4 natural-language prompts |
| **Consensus** | AI academic search engine | Freely available version at the time | Web interface, free tier | Semantic Scholar, OpenAlex and own web crawl, covering all of PubMed (~220M) | Hybrid semantic (embeddings) + keyword (BM25) search | Native retrieval over indexed scholarly corpus | Default; new conversation per prompt | 4 natural-language prompts |
| **Scite** | AI research assistant (citation-based) | Freely available version at the time | Web interface, free tier | Scite citation database (>185M publications and 1.3B citation statements) | Generative assistant over citation database (Smart Citations) | Citation database supplemented with the open web | Default; open-web source setting; new conversation per prompt | 4 natural-language prompts |

**Supplementary Table S1.** Technical characterization of the five evaluated literature search tools, summarizing for each tool its type, version, access mode, underlying corpus, retrieval strategy, web-retrieval capability, configuration, and query format. All tools were evaluated in their freely available versions between August 2025 and January 2026; the reported technical characteristics and corpus sizes are as stated by the respective vendors and were accessed on June 8, 2026. Abbreviations: BM25, Best Matching 25; MeSH, Medical Subject Headings; NLM/NCBI, National Library of Medicine / National Center for Biotechnology Information.

|  | **Prompt** | **Quadrant 1 : Developed & Broad Topic** | **Quadrant 2 : Developed & Specific Topic** | **Quadrant 3: Emerging & Specific Topic** | **Quadrant 4: Emerging & Broad Topic** |
| --- | --- | --- | --- | --- | --- |
| **Natural language tools** | **Research oriented** | How effective is cardiac resynchronization therapy by biventricular pacing in low ejection fraction heart failure with wide QRS? | How does multipoint ventricular pacing compare to biventricular pacing in improving outcomes of cardiac resynchronization therapy in heart failure patients with low ejection fraction and wide QRS? | How effective are artificial intelligence algorithms in predicting patient response to cardiac resynchronization therapy with biventricular pacing in heart failure patients with low ejection fraction and wide QRS complex? | How does the efficacy of left bundle branch or His-Purkinje conduction system pacing compare to biventricular pacing for cardiac resynchronization therapy in heart failure patients? |
|  | **Practice oriented** | What is the clinical benefit of cardiac resynchronization therapy by biventricular pacing in low ejection fraction heart failure with wide QRS? | In clinical practice, when should multipoint ventricular pacing be considered over biventricular pacing for cardiac resynchronization therapy in heart failure patients? | What is the clinical utility of using artificial intelligence to guide cardiac resynchronization therapy by biventricular pacing in patients with heart failure, low ejection fraction, and wide QRS? | What are the clinical benefits of left bundle branch or His-Purkinje conduction system pacing compared to biventricular pacing in managing heart failure through cardiac resynchronization therapy? |
|  | **General questioning** | What are the impacts of cardiac resynchronization therapy in heart failure? | What is the difference between multipoint and biventricular pacing in cardiac resynchronization therapy for heart failure? | What role does artificial intelligence play in optimizing outcomes of cardiac resynchronization therapy in heart failure with reduced ejection fraction and wide QRS? | What impact does conduction system pacing have on cardiac resynchronization therapy outcomes compared to traditional biventricular pacing in heart failure? |
|  | **Active form (task)** | Find me accurate references about effectiveness of cardiac resynchronization therapy by biventricular pacing in low ejection fraction heart failure with wide QRS | Find me key references about the effectiveness of multipoint ventricular pacing in comparison to biventricular pacing for cardiac resynchronization therapy in heart failure. | Find me accurate references about the effectiveness of artificial intelligence algorithms in predicting response to cardiac resynchronization therapy with biventricular pacing in heart failure patients with low ejection fraction and wide QRS. | Find me reliable and recent references comparing the efficacy of left bundle branch or His-Purkinje conduction system pacing with biventricular pacing for cardiac resynchronization therapy in heart failure. |
|  | **Prompt** | **Quadrant 1 : Developed & Broad Topic** | **Quadrant 2 : Developed & Specific Topic** | **Quadrant 3 : Emerging & Specific Topic** | **Quadrant 4 : Emerging & Broad Topic** |
| **Pubmed** | **Boolean** | ("cardiac resynchronization therapy") AND (("heart failure") OR ("cardiac failure")) | ((Clinical outcome) AND (Left Ventricular Multipoint Pacing) AND (Conventional Biventricular Pacing) AND (Cardiac Resynchronization Therapy)) | (Algorithms) AND (Cardiac Resynchronization therapy) | ((Left bundle branch pacing) AND (Biventricular pacing) AND (cardiac resynchronization therapy)) |
|  | **Expert** | ("cardiac resynchronization therapy" OR "biventricular pacing" OR "CRT") AND ("heart failure" OR "congestive heart failure") AND ("effectiveness" OR "efficacy" OR "outcome" OR "mortality") AND (("N Engl J Med"[Journal]) OR ("Lancet"[Journal]) OR ("JAMA"[Journal]) OR ("Circulation"[Journal]) OR ("Eur Heart J"[Journal]) OR ("J Am Coll Cardiol"[Journal])) AND ("1900/01/01"[Date - Publication] : "2006/12/31"[Date - Publication]) AND (randomized controlled trial[pt]) | ((Clinical outcome) OR (End Point) OR (Clinical Efficacy)) AND ((left ventricular multipoint pacing) OR (Multipoint Left Ventricular Stimulation) OR (Left Ventricular Multi-Site Pacing)) AND ((conventional biventricular pacing) OR (Standard Biventricular Pacing) OR (Biventricular Stimulation) OR (Resynchronization Pacing)) AND ((cardiac resynchronization therapy) OR (Biventricular pacing) OR (Atrio Biventricular Pacing)) | (Algorithms) AND ((Cardiac Resynchronization therapy) OR (Biventricular pacing) OR (Atrio Biventricular Pacing)) | ((Left bundle branch pacing) OR (Left-sided His-Purkinje system pacing) OR (Left Bundle Branch Area Pacing) OR (Left Bundle Branch Stimulation)) AND ((Biventricular pacing) OR (Biventricular Stimulation) OR (Resynchronization Pacing)) AND ((cardiac resynchronization therapy) OR (Biventricular pacing) OR (Atrio Biventricular Pacing)) |
|  | **Simple** | Cardiac resynchronization therapy heart failure effectiveness | Left bundle branch pacing versus biventricular pacing for cardiac resynchronization therapy | Algorithms cardiac resynchronization therapy | Left bundle branch pacing versus biventricular pacing for cardiac resynchronization therapy |

**Supplementary Table S2.** Prompt querying for articles retrieving, per topic.

| **Topic** | **SLR** | **Reference** | **DOI** |
| --- | --- | --- | --- |
| **A** | **"Cardiac Resynchronization Therapy for Patients with Left Ventricular Systolic Dysfunction: A Systematic Review"** | Auricchio A, Stellbrink C, Sack S, Block M, Vogt J, Bakker P, Huth C, Schöndube F, Wolfhard U, Böcker D, Krahnefeld O, Kirkels H; Pacing Therapies in Congestive Heart Failure (PATH-CHF) Study Group. Long-term clinical effect of hemodynamically optimized cardiac resynchronization therapy in patients with heart failure and ventricular conduction delay. J Am Coll Cardiol. 2002 Jun 19;39(12):2026-33. | 10.1016/S0735-1097(02)01895-8 |
|  |  | Cazeau S, Leclercq C, Lavergne T, Walker S, Varma C, Linde C, Garrigue S, Kappenberger L, Haywood GA, Santini M, Bailleul C, Daubert JC; Multisite Stimulation in Cardiomyopathies (MUSTIC) Study Investigators. Effects of multisite biventricular pacing in patients with heart failure and intraventricular conduction delay. N Engl J Med. 2001 Mar 22;344(12):873-80. | 10.1056/NEJM200103223441202 |
|  |  | Higgins SL, Hummel JD, Niazi IK, Giudici MC, Worley SJ, Saxon LA, Boehmer JP, Higginbotham MB, De Marco T, Foster E, Yong PG. Cardiac resynchronization therapy for the treatment of heart failure in patients with intraventricular conduction delay and malignant ventricular tachyarrhythmias. J Am Coll Cardiol. 2003 Oct 15;42(8):1454-9. | 10.1016/S0735-1097(03)01042-8 |
|  |  | Leclercq C, Walker S, Linde C, Clementy J, Marshall AJ, Ritter P, Djiane P, Mabo P, Levy T, Gadler F, Bailleul C, Daubert JC. Comparative effects of permanent biventricular and right-univentricular pacing in heart failure patients with chronic atrial fibrillation. Eur Heart J. 2002 Nov;23(22):1780-7. | 10.1053/EUHJ.2002.3232 |
|  |  | Leclercq C. Upgrading from right ventricular pacing to biventricular pacing in pacemaker patients with chronic heart failure. Heart. 1 janv 2008;94(1):102. | 10.1136/HRT.2005.065094 |
|  |  | Abraham WT, Fisher WG, Smith AL, Delurgio DB, Leon AR, Loh E, Kocovic DZ, Packer M, Clavell AL, Hayes DL, Ellestad M, Trupp RJ, Underwood J, Pickering F, Truex C, McAtee P, Messenger J; MIRACLE Study Group. Multicenter InSync Randomized Clinical Evaluation. Cardiac resynchronization in chronic heart failure. N Engl J Med. 2002 Jun 13;346(24):1845-53. | 10.1056/NEJMOA013168 |
|  |  | Bristow MR, Saxon LA, Boehmer J, Krueger S, Kass DA, De Marco T, Carson P, DiCarlo L, DeMets D, White BG, DeVries DW, Feldman AM; Comparison of Medical Therapy, Pacing, and Defibrillation in Heart Failure (COMPANION) Investigators. Cardiac-resynchronization therapy with or without an implantable defibrillator in advanced chronic heart failure. N Engl J Med. 2004 May 20;350(21):2140-50. | 10.1056/NEJMOA032423 |
|  |  | Young JB, Abraham WT, Smith AL, Leon AR, Lieberman R, Wilkoff B, Canby RC, Schroeder JS, Liem LB, Hall S, Wheelan K; Multicenter InSync ICD Randomized Clinical Evaluation (MIRACLE ICD) Trial Investigators. Combined cardiac resynchronization and implantable cardioversion defibrillation in advanced chronic heart failure: the MIRACLE ICD Trial. JAMA. 2003 May 28;289(20):2685-94. | 10.1001/JAMA.289.20.2685 |
|  |  | Abraham WT, Young JB, Leon AR, et al; Multicenter InSync ICD II Study Group. Effects of cardiac resynchronization on disease progression in patients with left ventricular systolic dysfunction, an indication for an implantable cardioverter-defibrillator, and mildly symptomatic chronic heart failure. Circulation. 2004;110:2864-2868. | 10.1161/01.CIR.0000146336.92331.D1 |
|  |  | Auricchio A, Stellbrink C, Butter C, et al; Pacing Therapies in Congestive Heart Failure II Study Group; Guidant Heart Failure Research Group. Clinical efficacy of cardiac resynchronization therapy using left ventricular pacing in heart failure patients stratified by severity of ventricular conduction delay. J Am Coll Cardiol. 2003;42:2109-2116. | 10.1016/J.JACC.2003.04.003 |
|  |  | Cleland JG, Daubert JC, Erdmann E, et al; Cardiac Resynchronization-Heart Failure (CARE-HF) Study Investigators. The effect of cardiac resynchronization on morbidity and mortality in heart failure. N Engl J Med. 2005;352:1539-1549. | 10.1056/NEJMOA050496 |
|  |  | Kindermann M, Hennen B, Jung J, Geisel J, Bohm M, Frohlig G. Biventricular versus conventional right ventricular stimulation for patients with standard pacing indication and left ventricular dysfunction: the Homburg Biventricular Pacing Evaluation (HOBIPACE). J Am Coll Cardiol. 2006;47:1927-1937. | 10.1016/J.JACC.2005.12.056 |
|  |  | US Food and Drug Administration. St Jude Medical Epic HF System including the Epic HF Model V-338 cardiac resynchronization therapy defibrillator, the Aescula LV model 1055K lead, the QuickSite LV model 1056K lead, and the model 3307, v4.5m programmer software, part 2: summary of safety and effectiveness [RHYTHM ICD]. 2005. http://www.fda.gov/cdrh/pdf3/P030054b.pdf. Accessed September 25, 2006. | - |
|  |  | US Food and Drug Administration. St Jude Medical Frontier model 5508L and Frontier II model 5586 Cardiac Resynchronization Therapy Pacemakers (CRT-P) supported on the model 3510 programmer platforms with the model 3307, v4.8m programmer software, part 2: summary of safety and effectiveness [VecTOR]. 2005. http://www.fda.gov/cdrh/pdf3/p030035s003b.pdf. Accessed September 25, 2006. | - |
| **Topic** | **SLR** | **Reference** | **DOI** |
| **B** | **"Clinical outcome of left ventricular multipoint pacing versus conventional biventricular pacing in cardiac resynchronization therapy: a systematic review and meta-analysis"** | BEHAR, J.M., BOSTOCK, J., ZHU LI, A.P., CHIN, H.M.S., JUBB, S., LENT, E., GAMBLE, J., FOLEY, P.W.X., BETTS, T.R., RINALDI, C.A. and HERRING, N. (2015), Cardiac Resynchronization Therapy Delivered Via a Multipolar Left Ventricular Lead is Associated with Reduced Mortality and Elimination of Phrenic Nerve Stimulation: Long-Term Follow-Up from a Multicenter Registry. J Cardiovasc Electrophysiol, 26: 540-546. | 10.1111/JCE.12625 |
|  |  | Giovanni B. Forleo, Luca Santini, Massimo Giammaria, Domenico Potenza, Antonio Curnis, Vito Calabrese, Danilo Ricciardi, Carlo D'agostino, Pasquale Notarstefano, Valentina Ribatti, Giovanni Morani, Massimo Mantica, Luigi Di Biase, Emanuele Bertaglia, Leonardo Calò, Francesco Zanon, Multipoint pacing via a quadripolar left-ventricular lead: preliminary results from the Italian registry on multipoint left-ventricular pacing in cardiac resynchronization therapy (IRON-MPP), EP Europace, Volume 19, Issue 7, July 2017, Pages 1170–1177 | 10.1093/EUROPACE/EUW094 |
|  |  | Giovanni B. Forleo, Luigi Di Biase, Rupinder Bharmi, Nirav Dalal, Germana Panattoni, Annalisa Pollastrelli, Manfredi Tesauro, Luca Santini, Andrea Natale, Francesco Romeo, Hospitalization rates and associated cost analysis of cardiac resynchronization therapy with an implantable defibrillator and quadripolar vs. bipolar left ventricular leads: a comparative effectiveness study, EP Europace, Volume 17, Issue 1, January 2015, Pages 101–107, | 10.1093/EUROPACE/EUU290 |
|  |  | Leshem, E., Suleiman, M., Laish-Farkash, A. et al. Impact of quadripolar LV leads on heart failure hospitalization rates among patients implanted with CRT-D: data from the Israeli ICD Registry. J Interv Card Electrophysiol 51, 5–12 (2018). | 10.1007/S10840-017-0305-Z |
|  |  | Gianluigi Bencardino, Antonio Di Monaco, Eleonora Russo, Cristian Colizzi, Francesco Perna, Gemma Pelargonio, Maria Lucia Narducci, Francesca Augusta Gabrielli, Gaetano Antonio Lanza, Antonio Giuseppe Rebuzzi, Filippo Crea, Outcome of Patients Treated by Cardiac Resynchronization Therapy Using a Quadripolar Left Ventricular Lead, Circulation Journal, 2016, Volume 80, Issue 3, Pages 613-618, Released on J-STAGE February 25, 2016, Advance online publication January 29, 2016, Online ISSN 1347-4820, Print ISSN 1346-9843 | 10.1253/CIRCJ.CJ-15-0932 |
|  |  | Gu, Min MD; Jin, Han MD; Hua, Wei MD, PhD*; Fan, Xiao-Han MD, PhD; Ding, Li-Gang MD, PhD; Wang, Jing MD, PhD; Niu, Hong-Xia MD, PhD; Cai, Chi MD; Zhang, Shu MD, PhD. Repetitive optimizing left ventricular pacing configurations with quadripolar leads improves response to cardiac resynchronization therapy: A single-center randomized clinical trial. Medicine 96(37):p e8066, September 2017. \| | 10.1097/MD.0000000000008066 |
|  |  | PAPPONE, C., ĆALOVIĆ, Ž., VICEDOMINI, G., CUKO, A., MCSPADDEN, L.C., RYU, K., ROMANO, E., BALDI, M., SAVIANO, M., PAPPONE, A., CIACCIO, C., GIANNELLI, L., IONESCU, B., PETRETTA, A., VITALE, R., FUNDALIOTIS, A., TAVAZZI, L. and SANTINELLI, V. (2015), Multipoint Left Ventricular Pacing in a Single Coronary Sinus Branch Improves Mid-Term Echocardiographic and Clinical Response to Cardiac Resynchronization Therapy. J Cardiovasc Electrophysiol, 26: 58-63. | 10.1111/JCE.12513 |
|  |  | Francesco Zanon, Lina Marcantoni, Enrico Baracca, Gianni Pastore, Daniela Lanza, Chiara Fraccaro, Claudio Picariello, Luca Conte, Silvio Aggio, Loris Roncon, Domenico Pacetta, Nima Badie, Franco Noventa, Frits W. Prinzen, Optimization of left ventricular pacing site plus multipoint pacing improves remodeling and clinical response to cardiac resynchronization therapy at 1 year, Heart Rhythm, Volume 13, Issue 8, 2016, Pages 1644-1651, ISSN 1547-5271 | 10.1016/J.HRTHM.2016.05.015 |
|  |  | Jonathan M. Behar, Hui Men Selina Chin, Steve Fearn, Julian O.M. Ormerod, James Gamble, Paul W.X. Foley, Julian Bostock, Simon Claridge, Tom Jackson, Manav Sohal, Antonios P. Antoniadis, Reza Razavi, Tim R. Betts, Neil Herring, Christopher Aldo Rinaldi, Cost-Effectiveness Analysis of Quadripolar Versus Bipolar Left Ventricular Leads for Cardiac Resynchronization Defibrillator Therapy in a Large, Multicenter UK Registry, JACC: Clinical Electrophysiology, Volume 3, Issue 2, 2017, Pages 107-116, ISSN 2405-500X, | 10.1016/J.JACEP.2016.04.009 |
|  |  | Leyva F, Zegard A, Qiu T, Acquaye E, Ferrante G, Walton J, et al. Cardiac Resynchronization Therapy Using Quadripolar Versus Non‐Quadripolar Left Ventricular Leads Programmed to Biventricular Pacing With Single‐Site Left Ventricular Pacing: Impact on Survival and Heart Failure Hospitalization. JAHA. 11 oct 2017;6(10):e007026. | 10.1161/JAHA.117.007026 |
|  |  | Mintu P. Turakhia, Michael Cao, Avi Fischer, Yelena Nabutovsky, Laurence S. Sloman, Nirav Dalal, Michael R. Gold, Reduced Mortality Associated With Quadripolar Compared to Bipolar Left Ventricular Leads in Cardiac Resynchronization Therapy, JACC: Clinical Electrophysiology, Volume 2, Issue 4, 2016 Pages 426-433, ISSN 2405-500X | 10.1016/J.JACEP.2016.02.007 |

| **Topic** | **SLR** | **Reference** | **DOI** |
| --- | --- | --- | --- |
| **C** | **"Artificial intelligence models in prediction of response to cardiac resynchronization therapy: a systematic review"** | Schmitz B, De Maria R, Gatsios D, Chrysanthakopoulou T, Landolina M, Gasparini M, Campolo J, Parolini M, Sanzo A, Galimberti P, Bianchi M, Lenders M, Brand E, Parodi O, Lunati M, Brand SM. Identification of genetic markers for treatment success in heart failure patients: insight from cardiac resynchronization therapy. Circ Cardiovasc Genet. 2014 Dec;7(6):760-70. | 10.1161/CIRCGENETICS.113.000384 |
|  |  | de A Fernandes F, Larsen K, He Z, Nascimento E, Peix A, Sha Q, Paez D, Garcia EV, Zhou W, Mesquita CT. A machine learning method integrating ECG and gated SPECT for cardiac resynchronization therapy decision support. Eur J Nucl Med Mol Imaging. 2023 Aug;50(10):3022-3033. | 10.1007/S00259-023-06259-4 |
|  |  | Galli E, Le Rolle V, Smiseth OA, Duchenne J, Aalen JM, Larsen CK, Sade EA, Hubert A, Anilkumar S, Penicka M, Linde C, Leclercq C, Hernandez A, Voigt JU, Donal E. Importance of Systematic Right Ventricular Assessment in Cardiac Resynchronization Therapy Candidates: A Machine Learning Approach. J Am Soc Echocardiogr. 2021 May;34(5):494-502. | 10.1016/J.ECHO.2020.12.025 |
|  |  | Hong JA, Lee SE, Kim SO, Kim MS, Lee HY, Cho HJ, Choi JO, Jeon ES, Hwang KK, Chae SC, Baek SH, Kang SM, Choi DJ, Yoo BS, Kim KH, Cho MC, Oh BH, Kim JJ. Determinants of left ventricular function improvement for cardiac resynchronization therapy candidates. ESC Heart Fail. 2022 Feb;9(1):283-292. | 10.1002/EHF2.13765 |
|  |  | Cikes M, Sanchez-Martinez S, Claggett B et al (2019) Machine learning-based phenogrouping in heart failure to identify responders to cardiac resynchronization therapy. Eur J Heart Fail 21:74–85. | 10.1002/EJHF.1333 |
|  |  | Field ME, Yu N, Wold N, Gold MR. Comparison of measures of ventricular delay on cardiac resynchronization therapy response. Heart Rhythm. 2020 Apr;17(4):615-620. | 10.1016/J.HRTHM.2019.11.023 |
|  |  | Haque A, Stubbs D, Hubig NC, Spinale FG, Richardson WJ. Interpretable machine learning predicts cardiac resynchronization therapy responses from personalized biochemical and biomechanical features. BMC Med Inform Decis Mak. 2022 Oct 31;22(1):282. | 10.1186/S12911-022-02015-0 |
|  |  | Howell S, Stivland TM, Stein K, Ellenbogen K, Tereshchenko LG. Response to cardiac resynchronisation therapy in men and women: a secondary analysis of the SMART-AV randomised controlled trial. BMJ Open. 2021 Oct 27;11(10):e049017. | 10.1136/BMJOPEN-2021-049017 |
|  |  | Kalscheur MM, Kipp RT, Tattersall MC, Mei C, Buhr KA, DeMets DL, Field ME, Eckhardt LL, Page CD. Machine Learning Algorithm Predicts Cardiac Resynchronization Therapy Outcomes: Lessons From the COMPANION Trial. Circ Arrhythm Electrophysiol. 2018 Jan;11(1):e005499. | 10.1161/CIRCEP.117.005499 |
|  |  | Bivona DJ, Tallavajhala S, Abdi M, Oomen PJA, Gao X, Malhotra R, Darby AE, Monfredi OJ, Mangrum JM, Mason PK, Mazimba S, Salerno M, Kramer CM, Epstein FH, Holmes JW, Bilchick KC. Machine learning for multidimensional response and survival after cardiac resynchronization therapy using features from cardiac magnetic resonance. Heart Rhythm O2. 2022 Jun 17;3(5):542-552. | 10.1016/J.HROO.2022.06.005 |
|  |  | Cai C, Tafti AP, Ngufor C, Zhang P, Xiao P, Dai M, Liu H, Noseworthy P, Chen M, Friedman PA, Cha YM. Using ensemble of ensemble machine learning methods to predict outcomes of cardiac resynchronization. J Cardiovasc Electrophysiol. 2021 Sep;32(9):2504-2514. doi: 10.1111/jce.15171. Epub 2021 Jul 27. PMID: 34260141. | 10.1111/JCE.15171 |
|  |  | Feeny AK, Rickard J, Patel D, Toro S, Trulock KM, Park CJ, LaBarbera MA, Varma N, Niebauer MJ, Sinha S, Gorodeski EZ, Grimm RA, Ji X, Barnard J, Madabhushi A, Spragg DD, Chung MK. Machine Learning Prediction of Response to Cardiac Resynchronization Therapy: Improvement Versus Current Guidelines. Circ Arrhythm Electrophysiol. 2019 Jul;12(7):e007316. | 10.1161/CIRCEP.119.007316 |
|  |  | Feeny AK, Rickard J, Trulock KM et al (2020) Machine learning of 12-lead QRS waveforms to identify cardiac resynchronization therapy patients with differential outcomes. Circ Arrhythm Electrophysiol 13:605–616. | 10.1161/CIRCEP.119.008210 |
|  |  | Gallard A, Bidaut A, Hubert A et al (2021) Characterization of responder profiles for cardiac resynchronization therapy through unsupervised clustering of clinical and strain data. JAm Soc Echocardiogr 34:483–493. https://doi.org/10.1016/j.echo.2021.01.019 | 10.1016/J.ECHO.2021.01.019 |
|  |  | Gallard A, Hubert A, Smiseth O, Voigt JU, Le Rolle V, Leclercq C, Bidaut A, Galli E, Donal E, Hernandez AI. Prediction of response to cardiac resynchronization therapy using a multi-feature learning method. Int J Cardiovasc Imaging. 2021 Mar;37(3):989-9980. | 10.1007/S10554-020-02083-1 |
|  |  | He Z, Zhang X, Zhao C, Ling X, Malhotra S, Qian Z, Wang Y, Hou X, Zou J, Zhou W. A method using deep learning to discover new predictors from left-ventricular mechanical dyssynchrony for CRT response. J Nucl Cardiol. 2023 Feb;30(1):201-213. | 10.1007/S12350-022-03067-5 |
|  |  | Hu SY, Santus E, Forsyth AW, Malhotra D, Haimson J, Chatterjee NA, Kramer DB, Barzilay R, Tulsky JA, Lindvall C. Can machine learning improve patient selection for cardiac resynchronization therapy? PLoS One. 2019 Oct 3;14(10):e0222397. | 10.1371/JOURNAL.PONE.0222397 |
|  |  | Lei J, Wang YG, Bhatta L, Ahmed J, Fan D, Wang J, Liu K. Ventricular geometry-regularized QRSd predicts cardiac resynchronization therapy response: machine learning from crosstalk between electrocardiography and echocardiography. Int J Cardiovasc Imaging. 2019 Jul;35(7):1221-1229. doi: 10.1007/s10554-019-01545-5. Epub 2019 May 18. | 10.1007/S10554-019-01545-5 |
|  |  | Liang Y, Ding R, Wang J, Gong X, Yu Z, Pan L, Huang J, Li R, Su Y, Zhu S, Ge J. Prediction of response after cardiac resynchronization therapy with machine learning. Int J Cardiol. 2021 Dec 1;344:120-126. doi: 10.1016/j.ijcard.2021.09.049. Epub 2021 Sep 28. | 10.1016/J.IJCARD.2021.09.049 |
|  |  | Riolet C, Menet A, Verdun S et al (2021) Clinical and prognostic implications of phenomapping in patients with heart failure receiving cardiac resynchronization therapy. Arch Cardiovasc Dis 114:197–210. | 10.1016/J.ACVD.2020.07.004 |
|  |  | Tokodi M, Schwertner WR, Kovács A, Tősér Z, Staub L, Sárkány A, Lakatos BK, Behon A, Boros AM, Perge P, Kutyifa V, Széplaki G, Gellér L, Merkely B, Kosztin A. Machine learning-based mortality prediction of patients undergoing cardiac resynchronization therapy: the SEMMELWEIS-CRT score. Eur Heart J. 2020 May 7;41(18):1747-1756. | 10.1093/EURHEARTJ/EHZ902 |
|  |  | Wouters PC, van de Leur RR, Vessies MB, van Stipdonk AMW, Ghossein MA, Hassink RJ, Doevendans PA, van der Harst P, Maass AH, Prinzen FW, Vernooy K, Meine M, van Es R. Electrocardiogram-based deep learning improves outcome prediction following cardiac resynchronization therapy. Eur Heart J. 2023 Feb 21;44(8):680-692. | 10.1093/EURHEARTJ/EHAC617 |

| **Topic** | **SLR** | **Reference** | **DOI** |
| --- | --- | --- | --- |
| **D** | **“Left bundle branch area pacing vs biventricular pacing for cardiac resynchronization: A systematic review and meta-analysis”** | Chen X, Ye Y, Wang Z, Jin Q, Qiu Z, Wang J, Qin S, Bai J, Wang W, Liang Y, Chen H, Sheng X, Gao F, Zhao X, Fu G, Ellenbogen KA, Su Y, Ge J. Cardiac resynchronization therapy via left bundle branch pacing vs. optimized biventricular pacing with adaptive algorithm in heart failure with left bundle branch block: a prospective, multi-centre, observational study. Europace. 2022 May 3;24(5):807-816. | 10.1093/EUROPACE/EUAB249 |
|  |  | Guo J, Li L, Xiao G, Ye T, Huang X, Meng F, Li Q, Chen S, Cai B. Remarkable response to cardiac resynchronization therapy via left bundle branch pacing in patients with true left bundle branch block. Clin Cardiol. 2020 Dec;43(12):1460-1468. | 10.1002/CLC.23462 |
|  |  | Hua J, Chen Y, Yu J, Xiong Q, Xia Z, Xia Z, Huang Q, Kong Q, Chen H, Zhang Y, Hu J, Li J, Hu J, Chen Q, Hong K. Long-term outcomes of left bundle branch area pacing versus biventricular pacing in patients with heart failure and complete left bundle branch block. Heart Vessels. 2022 Jul;37(7):1162-1174. | 10.1007/S00380-021-02016-5 |
|  |  | Li X, Qiu C, Xie R, Ma W, Wang Z, Li H, Wang H, Hua W, Zhang S, Yao Y, Fan X. Left bundle branch area pacing delivery of cardiac resynchronization therapy and comparison with biventricular pacing. ESC Heart Fail. 2020 Aug;7(4):1711-1722. | 10.1002/EHF2.12731 |
|  |  | Liu W, Hu C, Wang Y, Cheng Y, Zhao Y, Liu Y, Zheng S, Chen H, Shu X. Mechanical Synchrony and Myocardial Work in Heart Failure Patients With Left Bundle Branch Area Pacing and Comparison With Biventricular Pacing. Front Cardiovasc Med. 2021 Aug 20;8:727611. | 10.3389/FCVM.2021.727611 |
|  |  | Rademakers LM, van den Broek JLPM, Bracke FA. Left bundle branch pacing as an alternative to biventricular pacing for cardiac resynchronisation therapy. Neth Heart J. 2023 Apr;31(4):140-149. | 10.1007/S12471-022-01712-9 |
|  |  | Wang Y, Gu K, Qian Z, Hou X, Chen X, Qiu Y, Jiang Z, Zhang X, Wu H, Chen M, Zou J. The efficacy of left bundle branch area pacing compared with biventricular pacing in patients with heart failure: A matched case-control study. J Cardiovasc Electrophysiol. 2020 Aug;31(8):2068-2077. | 10.1111/JCE.14628 |
|  |  | Wu S, Su L, Vijayaraman P, Zheng R, Cai M, Xu L, Shi R, Huang Z, Whinnett ZI, Huang W. Left Bundle Branch Pacing for Cardiac Resynchronization Therapy: Nonrandomized On-Treatment Comparison With His Bundle Pacing and Biventricular Pacing. Can J Cardiol. 2021 Feb;37(2):319-328. | 10.1016/J.CJCA.2020.04.037 |
|  |  | Wang Y, Zhu H, Hou X, Wang Z, Zou F, Qian Z, Wei Y, Wang X, Zhang L, Li X, Liu Z, Xue S, Qin C, Zeng J, Li H, Wu H, Ma H, Ellenbogen KA, Gold MR, Fan X, Zou J; LBBP-RESYNC Investigators. Randomized Trial of Left Bundle Branch vs Biventricular Pacing for Cardiac Resynchronization Therapy. J Am Coll Cardiol. 2022 Sep 27;80(13):1205-1216. | 10.1016/J.JACC.2022.07.019 |
|  |  | Ezzeddine FM, Pistiolis SM, Pujol-Lopez M, Lavelle M, Wan EY, Patton KK, Robinson M, Lador A, Tamirisa K, Karim S, Linde C, Parkash R, Birgersdotter-Green U, Russo AM, Chung M, Cha YM. Outcomes of conduction system pacing for cardiac resynchronization therapy in patients with heart failure: A multicenter experience. Heart Rhythm. 2023 Jun;20(6):863-871. | 10.1016/J.HRTHM.2023.02.018 |
|  |  | Liang Y, Xiao Z, Liu X, Wang J, Yu Z, Gong X, Lu H, Yang S, Gu M, Zhang L, Li M, Pan L, Li X, Chen X, Su Y, Hua W, Ge J. Left Bundle Branch Area Pacing versus Biventricular Pacing for Cardiac Resynchronization Therapy on Morbidity and Mortality. Cardiovasc Drugs Ther. 2024 Jun;38(3):471-481. | 10.1007/S10557-022-07410-3 |
|  |  | Vijayaraman P, Sharma PS, Cano Ó, Ponnusamy SS, Herweg B, Zanon F, Jastrzebski M, Zou J, Chelu MG, Vernooy K, Whinnett ZI, Nair GM, Molina-Lerma M, Curila K, Zalavadia D, Haseeb A, Dye C, Vipparthy SC, Brunetti R, Moskal P, Ross A, van Stipdonk A, George J, Qadeer YK, Mumtaz M, Kolominsky J, Zahra SA, Golian M, Marcantoni L, Subzposh FA, Ellenbogen KA. Comparison of Left Bundle Branch Area Pacing and Biventricular Pacing in Candidates for Resynchronization Therapy. J Am Coll Cardiol. 2023 Jul 18;82(3):228-241. | 10.1016/J.JACC.2023.05.006 |

**Supplementary Table S3.** List of the articles extracted from SLRs per topic.

| **Variable** | **Beta (CI 95%)** | **p-value** |
| --- | --- | --- |
| **N =** | 75 |  |
| **(Intercept)** | 38 (22 - 53) | <0.001 |
| **PubMed** | - | - |
| **ChatGPT** | 32 (15 ; 49) | <0.001 |
| **Consensus** | -5 ( -22 ;12) | 0.553 |
| **Elicit** | 5 ( -12 ; 22) | 0.569 |
| **Scite** | -31 ( -48 ; -13) | <0.001 |
| **Topic C** | - | - |
| **Topic A** | 30 (16 ;44) | <0.001 |
| **Topic B** | 18 (4 ;33) | 0.013 |
| **Topic D** | 16 (2 ;30) | 0.024 |
| **BIC** | 705 |  |

**Supplementary Table S4.** Beta coefficients with confidence interval and p-values of independent variables associated with the percentage of relevant references (dependent variable). *Abbreviations: BIC, Bayesian Information Criterion; CI, confidence interval; N, Number.*

| **Variable** | **Beta (CI 95%)** | **p-value** |
| --- | --- | --- |
| **N =** | 75 |  |
| **(Intercept)** | 33 (19 ; 46) | <0.001 |
| **PubMed** | - | - |
| **ChatGPT** | 33 (19 ; 47) | <0.001 |
| **Consensus** | 3 ( -11 ;17) | 0.642 |
| **Elicit** | 12 ( -1 ;26) | 0.079 |
| **Scite** | -9 ( -23 ;5) | 0.183 |
| **Topic B** | - | - |
| **Topic A** | -15 ( -27 ; -3) | 0.013 |
| **Topic C** | -13 ( -24 ; -1) | 0.034 |
| **Topic D** | -12 ( -24 ; -0.2) | 0.046 |
| **BIC** | 675 |  |

**Supplementary Table S5.** Beta coefficients with confidence interval and p-values of independent variables associated with the percentage of key reference (dependent variable). *Abbreviations: BIC, Bayesian Information Criterion; CI, confidence interval; N, Number.*

**Supplementary Figures**

**Supplementary Figure S1. Complete methodological workflow of the study; detailed version of Figure 1.** This expanded diagram makes explicit the two sources feeding the pooled corpus, the 59 primary references of the high-quality systematic literature reviews (SLRs) and the references returned by the 76 tool queries, and, through the crossing arrows, the contribution of each source to the two expert-defined gold standards. All other elements (tools, standardized queries, retrieval, and blinded expert evaluation) are as defined in the Figure 1 legend and the Methods.

**Supplementary Figure S2.** Percentage of relevant articles retrieved by AI-based and conventional tools, per prompt.

**Supplementary Figure S3.** Percentage of articles retrieved by AI-based and conventional tools included in Key References by the experts, per prompt.
